# Trace element contamination differentiates the natural population of Scots pine: evidence from DNA microsatellites and needle morphology

**DOI:** 10.1007/s11356-016-7472-9

**Published:** 2016-08-20

**Authors:** Ewa Chudzińska, Konrad Celiński, Ewa M. Pawlaczyk, Aleksandra Wojnicka-Półtorak, Jean B. Diatta

**Affiliations:** 1 Faculty of Biology, Department of Genetics, Adam Mickiewicz University in Poznań, Umultowska 89, 61-614 Poznań, Poland; 2 Department of Agricultural Chemistry and Environmental Biogeochemistry, Poznań University of Life Sciences, Wojska Polskiego 71F, 60-625 Poznań, Poland

**Keywords:** Trace elements, Mg, Fe, *Pinus sylvestris*, Fluctuating asymmetry, Genetic diversity, Microsatellites

## Abstract

The Scots pine is often used in the biomonitoring of forests. Studies on the chemical composition plus variability of its needles morphological structure allow for an assessment of the state of environmental pollution. However, in their natural populations, the response of individual trees to stress differs. This study reports on the influence of long-term soil contamination with trace elements on the morphology of the needles, its possible relation to the differentiation of the genetic pool, and their implications for biomonitoring. In the natural and self-renewable pine stand growing near the point polluter (zinc smelter, Upper Silesia, Poland), two categories of trees are observed with respect to their health status: pollution-tolerant (T) and pollution-sensitive (S). A detailed analysis of the trace element content of the needles reveals that the concentration of Cd, Zn, Pb, and Cu in the needles is significantly higher in S as compared to T individuals. The metal accumulation pattern decidedly follows the sequence Pb > Cd > Cu > Zn. An analysis of the fluctuating asymmetry (FA) of the needles reveals that sensitive trees showed an FA index ten times higher in comparison to tolerant ones. Moreover, the high differences between these S and T tree groups are also observed in the basic genetic diversity parameters investigated by an analysis of DNA simple sequence repeats (SSR). The concentration of trace elements in pine needles, distinct in sensitive and tolerant trees and in connection with their morphological and genetic characteristics, may reflect an adaptation process. The level of Mg and Fe content in the needles could be a physiological-toxicological index for evaluating trace element “lethality” expressed as Mg and Fe mineral-survival strategies. The example of differences described in this Scots pine population should be taken into consideration in ecotoxicological research to better interpret the obtained results.

## Introduction

In ecotoxicological studies, in order to establish the relationship between pollution and the reaction caused on the population level, the analysis of bioindicators is an essential element; however, for the effective application of biomarkers, their main traits must be known. By using a variety of methods (e.g., analytical, morphological, and molecular), the evaluation of the environmental risks offers researchers the ability to predict what might happen to the local population in the near future. However, the well-known individual variability of each specimen’s response must be taken into consideration when interpreting the results. The Scots pine (*Pinus sylvestris* L.), the main component of the coniferous forests of Europe, is a species commonly used for the passive biomonitoring of environmental pollution (Pöykiö et al. 2010; Pietrzykowski et al. 2014; Świercz et al. 2014).

In particular, its pine needles, due to their high sensitivity to many kinds of pollutants, are an important bio-indicator available in the Environmental Sample Banks and are collected in order to obtain information about the ecosystem (Markert et al., 2014). The highest diagnostic value in the evaluation of forest environmental pollution is attributed to 2-year-old pine needles because they are fully formed, and the content of the analyzed elements within them is most stable (ICP Forests, 2010). Trace metals (i.e. Cd, Zn, Pb, and Cu) are identified among environmental pollutants as toxic elements to nearly all living organisms (WHO 2010). A large amount of data shows a significant negative relationship between genetic diversity and metal concentration (Deng et al. 2007; Minkowska et al. 2014; Chudzińska 2013). Soil contaminations often cause genetic erosion and reduce the size of the population (Ribeiro and Lopes 2013; Chudzińska et al. 2014a). Heavy pollution can lead to the disappearance of local species, and to foreign plants taking their place. Therefore, studies on the relationship between trace metals exposure, fitness, and the population diversity (using various markers) of native populations have been progressively conducted.

In the case of *P. sylvestris*, a simple and very effective method to assess the health status of the tree is fluctuating asymmetry (FA). As stated by definition, FA represents small, non-directional deviations from perfect symmetry in morphological characters. This is the result of stress acting on the specimens under development (Van Dongen 2006). According to meta-analyses conducted by Beasley et al. (2013), “the use of FA as a biomarker of environmental stress is a legitimate tool particularly when studies verify the biological relevance of stressors for the study organism.” Due to their double-needled fascicles, pine needles are a convenient object for testing this parameter (Kozlov et al. 2002; De Coster et al. 2013; Makarenko et al. 2016). Deviation from needle symmetry, as a result of a disruption of its proper growth, may be caused under stress conditions by the allocation of resources (in morphogenesis) from developmental quality to growth. Therefore, a significant increase of the FA value observed among pine trees growing in the heavily contaminated soil is an index of environmental and genetic stress (Leamy and Klingenberg 2005; Chudzińska et al. 2014b).

Microsatellite DNA markers, also known as simple sequence repeats (SSR) due to their very substantial variability and often non-neutral character, is increasingly used to assess the genetic variation of natural populations growing under strong stress conditions (Rocha et al. 2002; Badri et al. 2008; Meyer et al. 2010). The frequency of mutations at SSRs depends on numerous factors including the region repeated, gene locus, and the species, age, and gender of a plant. Estimates of mutation rates vary from 10^2^ to 10^6^ (Li et al. 2002 and literature cited within). Two mechanisms can be invoked to explain the high mutation rates at SSRs: slippage during DNA replication and homologous recombination between DNA strands. The efficiency of the two mechanisms is likely to depend on environmental conditions, so microsatellite markers may be useful in studying the adaptive responses of plants to stress. These markers are described as over-represented in the stress response genes of microbial organisms (Rocha et al. 2002). There is increasing evidence that SSRs can play a functional role in gene expression as important sources of adaptive genetic variation, both within and between species (Geng et al. 2015). An analysis of the diversity of SSRs is a suitable method in this context, but should be complemented by other techniques, e.g., by measuring the activity of enzymes involved in trace element stress response. If metals can lead to alterations in the genetic pool, the genetic parameters can be used in the assessment of the impacts of pollution in natural systems.

This work focused on examining the biological effects of soil contamination for pine trees using needle indices as endpoints in the context of population differentiation, and its consequences for ecotoxicological research. The objectives of this paper are (1) an investigation of the developmental instability (expressed through FA index) of trees growing at an area chronically exposed to trace metals pollution; (2) a determination of the basic population-genetic parameters using microsatellite DNA markers in order to characterize the genetic pool of the population; (3) the verification of the hypothesis, that the concentrations of Mg and Fe in Scots pine needles could be a physiological-toxicological index of trace metals “lethality”; and (4) an application of data from sensitive and tolerant pine trees for biomonitoring purposes.

## Materials and methods

### Plant and soil sampling and chemical analyses

The study was carried out on the site close to the zinc and lead plant at Miasteczko Śląskie, South Poland. In this study, C + 1 old needles (i.e., needles from the current year plus one) of *P. sylvestris* were used as bioindicators in determining the soil pollution around the point polluter at Miasteczko Śląskie, South Poland. Trees were growing in a strongly impacted area located 500–1000 m north–northeast of the point polluter. Needle pairs were collected in late summer at intervals of 4 years (2006–2010–2014) in a natural population according to the recommendations of the ICP Manual Forest (ICP Forests 2010). The analyzed trees were divided into two groups evaluated on the basis of the percent of needle discoloration, crown defoliation, presence of C + 1 old needles, and seed germinability (Table 1). Individuals in good condition and exhibiting no major signs of damage were designated as the pollution-tolerant group (T), while individuals with needle discoloration and considerable crown damage as the pollution-sensitive group (S). Each group of trees was represented by 33 individuals growing in a middle-aged (40–50 years old) Scots pine stand on a sandy, arid, relatively nutrient-poor soil. The same trees were sampled over the years.

**Table 1:** Criteria of damage in collected trees of Scots pine growing in heavy metal polluted soil (after Chudzińska et al. 2014a, b)

Category	Needle discoloration (%)	Crown defoliation (%)	Needle damage (%)	Presence of C + 1 old^a^ needles (%)	Seed germinability (%)
Sensitive (S)	>60	>60	>51	<30	<50
Tolerant (T)	>25	26–60	11–50	30–90	50–90

^a^ i.e. the current-plus 1-year needles

Detailed information on soil sampling and analyses are described in earlier works (Diatta et al. 2008, 2011; Chudzińska et al. 2014a). Soil samples were collected at three depths, i.e., 0–20, 20–40, 40–60, and 60–80 cm. The aqua regia soluble metal (i.e., Cu, Zn, Pb, Cd) concentrations were determined according to the ISO 11466 (ISO 11466 1995) procedure by means of the AAS method (Varian 250 Spectra plus) and considered as total metal content. Bioavailable metal forms were extracted by applying 0.10 mol NaNO_3_ dm^−3^ (Gupta and Aten 1993). Prior to grinding, needle samples were dried at 50 °C for 3 days. A 0.250 ± 0.001 g portion of ground needles was weighed into tubes and mineralized in concentrated HNO_3_ (MARS5 CEM Corporation). The digests were then filtered, and the trace elements were determined by the AAS method. Analyses were performed in duplicate (Diatta et al. 2008, 2011).

### Biometric methods

From each of the 198 specimens (33 trees from each analyzed year, from 2 groups: sensitive and tolerant), 10 shoots were collected and 3960 needles were measured. Measurements (made twice by two independent persons) were taken under a stereomicroscope to the nearest 0.025 mm, next to the directional asymmetry. Anti-symmetry and trait size were checked according to the protocol described by Kozlov and Niemelä (1999) and Kozlov et al. (2002). The length of the two needles in a shoot was measured and the difference between the needles was estimated as an absolute value (left minus right needle length) between needles in shoots. The FA index was calculated as the ratio between the difference in the needle length and the average needle length of the two needles in a pair (e.g., Kozlov et al. 2002; Chudzińska et al. 2014b). Two independent measurements of the absolute difference in the length of the two needles generated similar results (*r* = 0.86, *n* = 1980, *p* < 0.001), indicating high repeatability.

### DNA extraction and SSR amplification

The genomic DNA was extracted using a modified cetyltrimethylammonium bromide (CTAB) protocol (Doyle and Doyle 1990), and dissolved in 0.1 × TE buffer (10 mM pH 8.0 Tris-HCl; 1 mM pH 8.0 EDTA) for subsequent use. The quality and quantity of the extracted DNA were measured on a Nanodrop™ ND-1000 (ThermoScientific) spectrophotometer and diluted to a final concentration of 20 ng/μl. Until amplification, DNA samples were stored at −20 and −4 °C for long-term and short-term storage, respectively.

For microsatellite genotyping, a total of six loci were initially tested for the presence of amplification products. Four of these loci—SPAG 7.14, SPAC 11.4 (Soranzo et al. 1998) and PtTX 4001, PtTX 4011 (Auckland et al. 2002)—produced the most successful results and were used for the analysis of all samples. The reverse primer of each primer pair was labeled with fluorescent 6FAM, PET, and VIC dyes (Applied Biosystems^©^). Amplifications were carried out separately for each marker in a 2720 Thermal Cycler (Applied Biosystems^©^) according to the protocol described by Celiński et al. (2013). Amplification products from all 120 individuals were separated with the 3130xl Genetic Analyzer (Applied Biosystems^©^) capillary electrophoresis system with GeneScan™600LIZ™ as an internal size standard. Individuals were analyzed and genotyped with the GeneMapper version 3.7 software (Applied Biosystems^©^).

### Statistical analyses

Biometric data were statistically analyzed using StatSoft Statistica (StatSoft Inc., Tulsa, OK, USA). The arithmetic mean and standard error of mean were calculated. The Shapiro-Wilk test did not show the normal distribution of the FA index. Then, the non-parametric analysis of variance (Kruskal-Wallis) and Wilcoxon test were conducted in order to determine whether statistically significant differences, in terms of the fluctuating asymmetry factor and trace element concentration in needles, occur between groups of trees (Ferguson and Takane 2007). Furthermore, the Spearman rank correlation coefficients between the FA index, trace element content, and observed heterozygosity were estimated. To provide a graphical presentation of the differentiation of the groups studied, the principal coordinate’s analysis (PCoA) and cluster analysis were made.

Genetic diversity and differentiation were analyzed using POPGENE version 1.31 (Yeh and Boyle 1997) and GenAIEx v.6.4 (Genetic Analysis In Excel; Peakall and Smouse 2012). The following genetic diversity parameters were calculated: the number of alleles (*N*_A_), the effective number of alleles (*N*_E_), the number of private alleles (*N*_AP_), the heterozygosity observed (*H*_O_) and expected (*H*_E_), the heterozygosity individual for particular samples (*H*_ind_), the fixation index (*F*), the Hardy-Weinberg equilibrium test (HWE), and the genetic differentiation (ϕ_PT_). In order to partition the total level of genotypic variance within and among the sensitive (S) and tolerant (T) groups of Scots pine trees, the hierarchical analysis of molecular variance (AMOVA) was calculated using GenAIEx v.6.5 (Genetic Analysis in Excel; Peakall and Smouse 2012). The significance of the variance components and the pairwise genetic differentiation was tested using 999 permutations.

## Results

### Trace metal, Mg, and Fe contents in soil and pine needles

For the content of metals in the soil at a depth of 0–20 cm, the mean values were 322.7, 386.0, 4.79, and 39.6 mg kg^−1^, respectively. The highest contents observed mainly consisted of zinc, lead, and cadmium, which is in line with data reported earlier by Chudzińska et al. (2014a). The assessment of the contamination state on the basis of threshold values for forest soils (Directive of the Polish Minister of Environment: Dz.U. no. 165, 2002) has shown that the contents of Zn, Pb, and Cd were significantly exceeded. The analysis of bioavailable metal forms (Gupta and Hani 1989) revealed that the bioavailable levels of Zn and Cd significantly exceeded threshold values, as was also observed for Pb, but only for the 0–20 cm layer. The data reported in Table 2 are sets of trace metals, Mg, and Fe contents in the C + 1 old pine needles during the 3 years of investigations (2006, 2010, and 2014; mean values). The range of Zn, Pb, and Cd consequently imply that the main pollution involved these metals, irrespective of tree group, i.e., tolerant (T) or sensitive (S). A decrease in all metal concentrations was observed, as compared to Mg and Fe, which exhibited an increase over time. Therefore, we conceptually designed two groups of mineral elements as key factors for Scots pine growth under metallurgical pollution, i.e., (i) Cu, Zn, Pb, Cd, and (ii) Mg, Fe. Mean values (Table 2) have shown the highest potential of pine sensitivity for metal accumulation, as the Δ-based mean values follows the sequence (mg kg^−1^): Pb (302.6) > Zn (95.3) > Cd (6.6) > Cu (5.6). The magnitude of metal pollution was evaluated by a comparison with the reference values (RefV) representing 10, 50, 1.0, and 0.05 mg kg^−1^ for Cu, Zn, Pb, and Cd, respectively. Only the RefV for Cu fluctuates within mean concentrations in tolerant (14.5 mg kg^−1^) and sensitive (8.9 mg kg^−1^) pine needles. This implies that Cu was not a mineral “stressor” impacting the normal growth of the investigated trees. Most striking were the concentrations of Mg as well as Fe, both slightly higher than the lowest values of their ranges: 300–1400 and 100–850 mg kg^−1^, respectively. Such levels reveal the response of these pines to the negative impact of metals; in particular, to Pb, Zn, and Cd, with visibly higher concentrations of Mg and Fe in the S trees as compared to the T trees.

**Table 2:** The mean concentration of trace metals, magnesium, and iron contents (mg/kg^−1^) in C + 1 old sensitive (S) and tolerant (T) Scots pine needles

Group	Year		Cu	Zn	Pb	Cd	Mg	Fe
S	2006	Mean	12.46	392.44	462.61	17.81	339.44	67.77
		SD	33.15	93.77	88.43	9.02	92.19	31.48
	2010	Mean	8.69	495.36	705.35	20.63	326.04	69.69
		SD	3.02	135.06	353.95	10.97	94.19	28.59
	2014	Mean	6.28	76.36	6.66	0.99	280.06	63.56
		SD	1.02	41.12	1.12	0.14	43.53	11.01
	All years	Min	4.3	35.3	5.5	0.8	184.0	25.6
		Max	27.9	895.8	1933.8	61.5	923.1	157.4
	Total	Grand mean	14.5	437.6	560.4	16.4	315.4	67.6
		SD	4.2	181.2	417.8	12.8	162.7	28.0
T	2006	Mean	11.98	419.57	425.74	11.74	335.11	64.49
		SD	1.80	55.94	131.16	3.91	75.54	26.11
	2010	Mean	7.10	570.95	513.89	15.35	282.70	79.31
		SD	2.92	207.72	258.65	7.65	95.26	30.38
	2014	Mean	10.02	169.40	6.55	0.976	278.04	58.90
		SD	2.32	91.95	1.74	0.19	52.15	32.73
	All years	Min	3.1	99.2	5.4	0.82	192.8	40.8
		Max	14.8	952.1	993.6	36.1	1188.2	1016.0
	Total	Grand mean	8.9	342.3	257.8	9.8	284.2	59.4
		SD	3.3	221.2	272.9	7.9	236.5	160.6
Δ (mean value basis) mg/kg			5.6	95.3	302.6	6.6	31.2	8.2
Accumulation % (sensitive over tolerant)			62.9	27.8	117.4	67.3	11.0	13.8
References^a,b^			Cu	Zn	Pb	Cd	Ranges^c^ for Mg	Ranges^c^ for Fe
Values (mg/kg)			10	50	1.0	0.05	300–1400	100–850

*SD* standard deviation

^a^Markert (1994)

^b^Pais and Benton Jones (1997)

^c^Białobok et al. (1993)

**Fig. 1:**
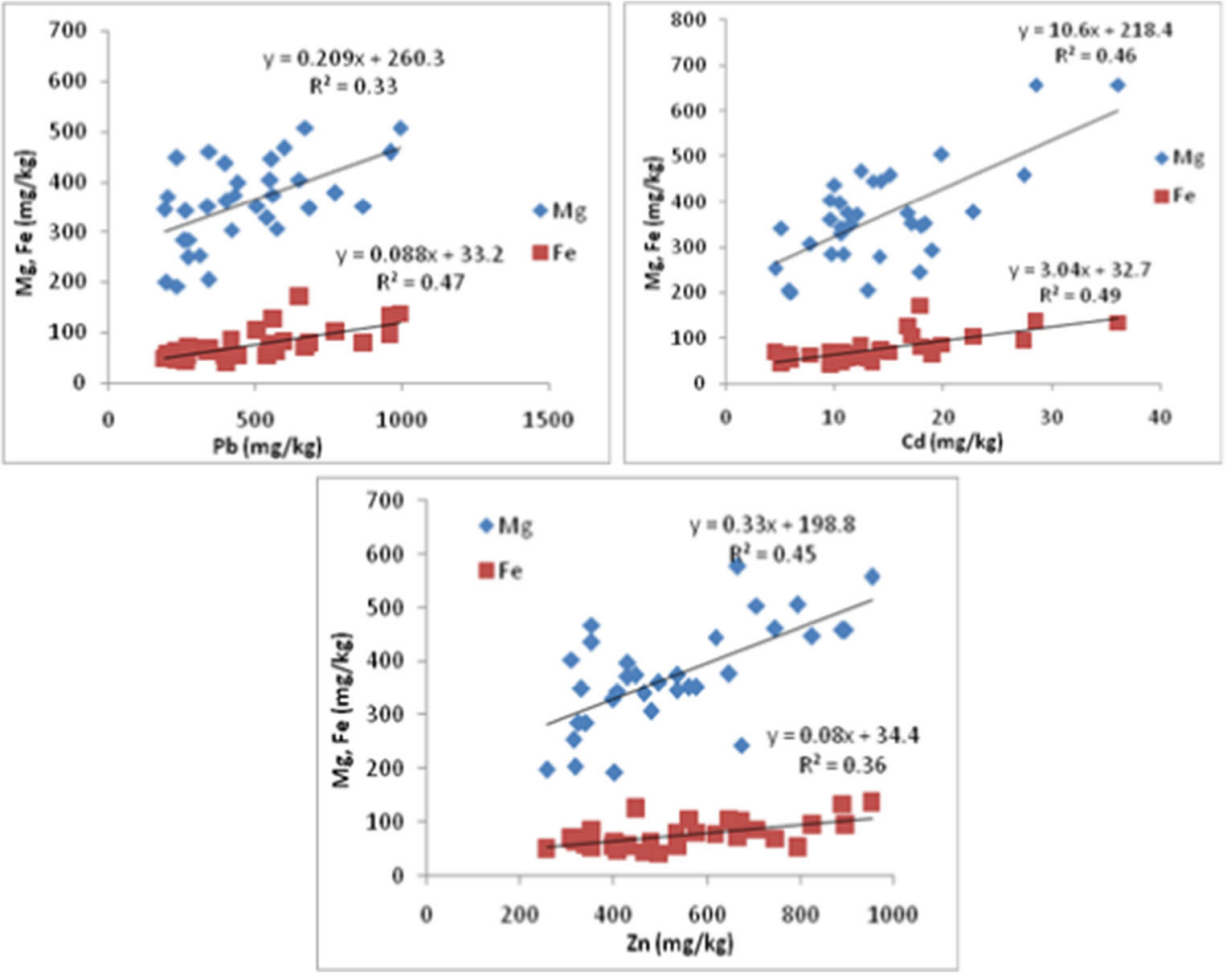
Magnesium and iron accumulation as influenced by Pb, Cd, and Zn concentrations in C + 1 old tolerant Scots pine needles. *R* ^2^—coefficient of determinations

**Fig. 2:**
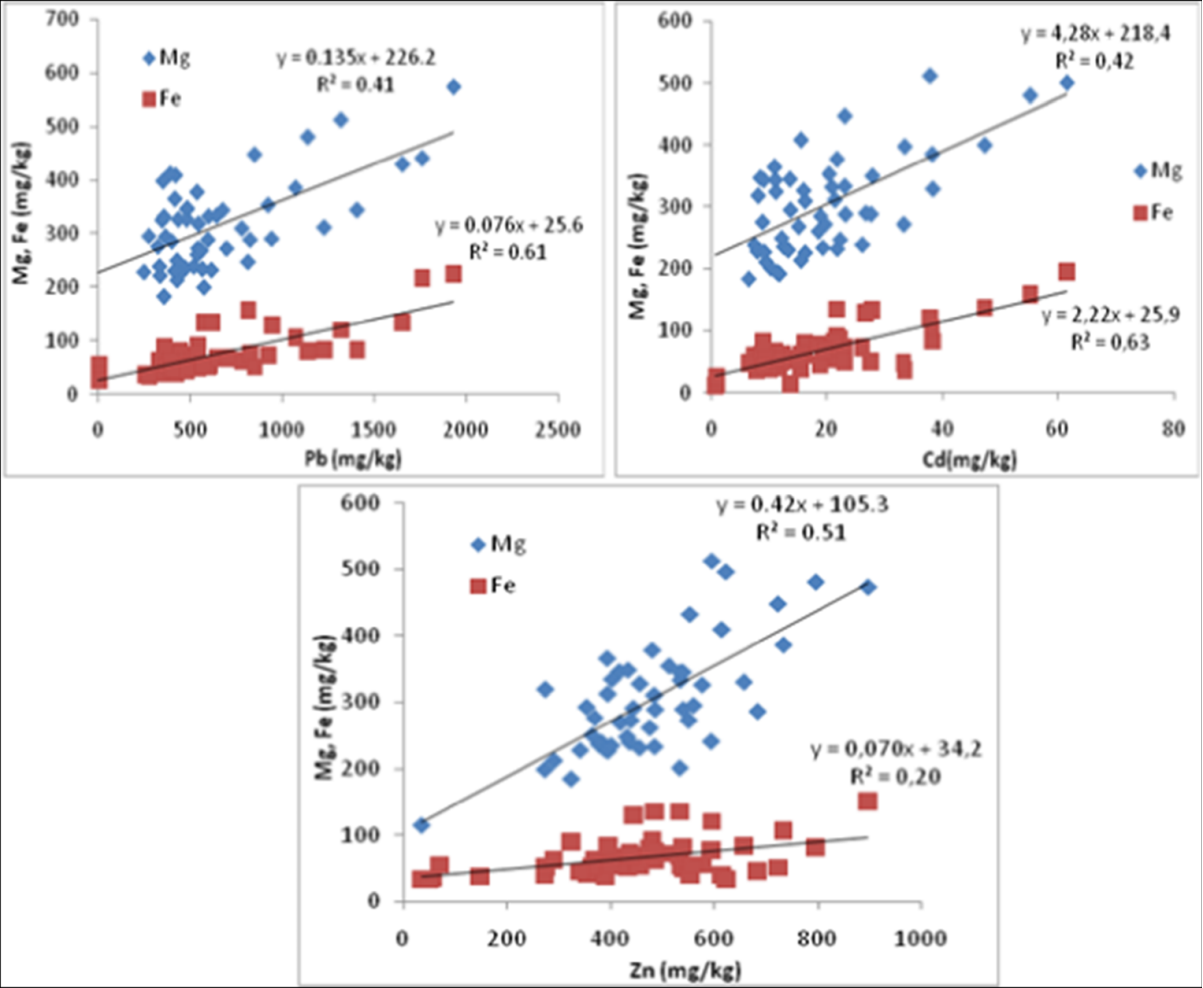
Magnesium and iron accumulation as influenced by Pb, Cd, and Zn concentrations in C + 1 old sensitive Scots pine needles. *R* ^2^—coefficient of determinations

The relationships reported in Fig. 1 (for tolerant Scots pine trees) and Fig. 2 (for sensitive Scots pine trees) show some discrepancies in terms of potential reactions—responses (R-R) of both pines. The higher the Zn, Pb, and Cd concentrations in the needles, the higher the level of Mg and Fe accumulation, irrespective of pine group. Two operational R-R ranges may be observed: (i) Mg with the highest *R* ^2^ values particularly for Cd and Zn, and (ii) Fe with the highest *R* ^2^ values particularly for Pb and Cd. Both groups, i.e., tolerant as well as sensitive, have exhibited responses to Cd that are indicative of the acute toxicity of this metal.

### Fluctuating asymmetry

**Fig. 3:**
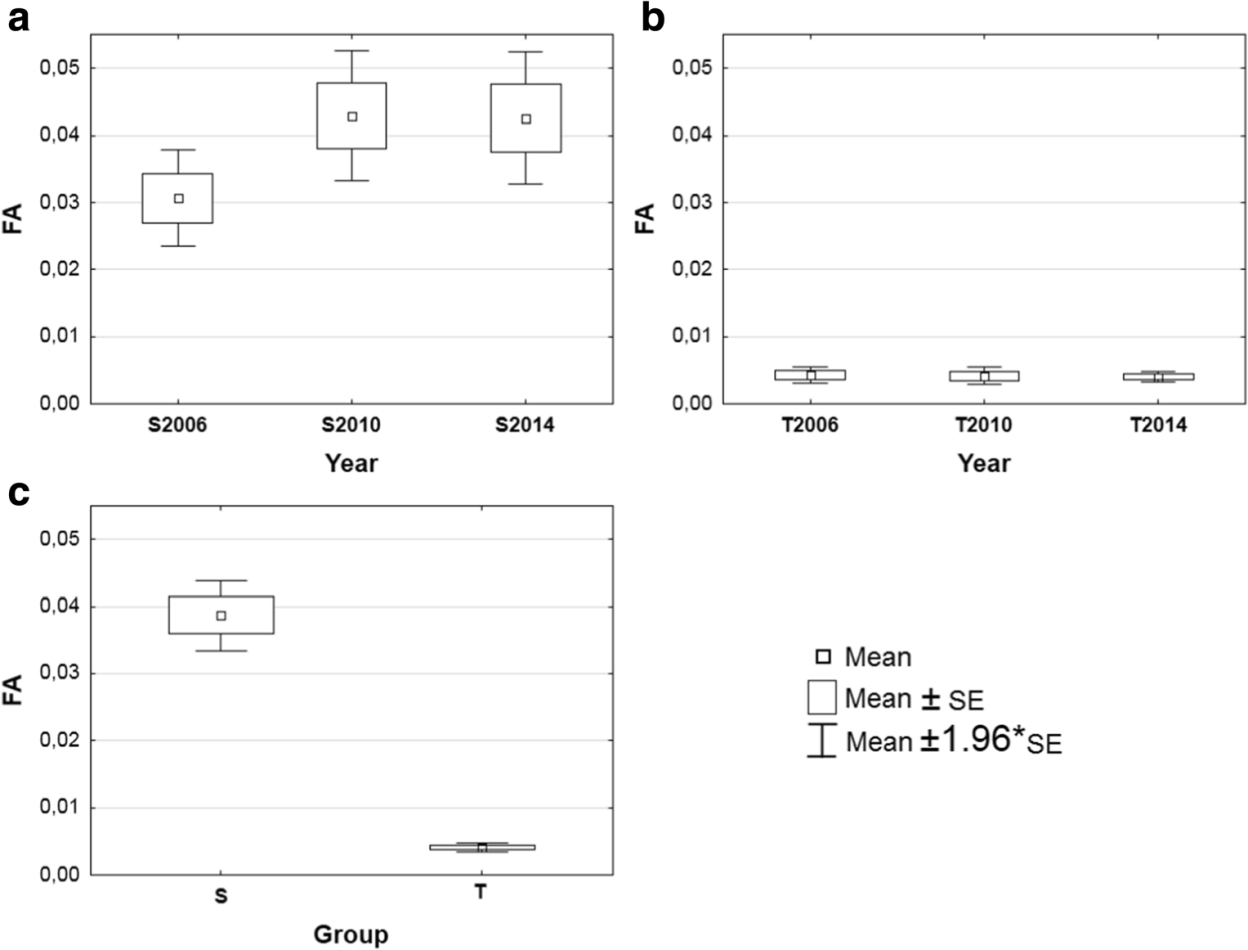
The box and whisker plot of the FA index for S (**a**) and T (**b**) groups of C + 1 old Scots pine needles in three time periods, and in total (**c**)

The length differences between left and right needles in the fascicle (Student *t* test) were significant (*t* = 7.25, df = 1979, *p* = 0.000), suggesting the occurrence of asymmetry. An absence of anti-symmetry was revealed by the normality of the distribution of left minus right needle values (*p* = 0.19), while the zero mean value (*p* = 0.17) confirmed the absence of directional asymmetry. Thus, the observed asymmetry was classified as fluctuating asymmetry (FA) (e.g., Kozlov and Niemelä 1999; Kozlov et al. 2002). The value of FA ranges from 0.0039 to 0.0043 (mean 0.0041) in the tolerant group (T) and from 0.0306 to 0.0429 (mean 0.0387) in the sensitive group (S). In the T group, the highest FA value was in the year 2006 and the lowest in the year 2014. In the sensitive group, the highest FA was in the year 2010 (the accumulation of trace metals such as zinc, lead, cadmium, and iron in needles was also the highest in this year) and the lowest in the year 2006. Pines in group S showed an FA index ten times greater than those in group T (Fig. 3). Differences between groups S and T in three consecutive time periods were statistically confirmed by the non-parametric Mann-Whitney *U* test (Table 3). In every studied year, as well as in total, the differences were highly statistically significant (*p* = 0.000). Within groups between particular years, the Friedman ANOVA for S (*χ* ^2^_(2;96)_ = 2.46; *p* = 0.293) as well as for T (*χ* ^2^_(2;96)_ = 2.82; *p* = 0.244) did not show any significant differences (Table 3). Spearman’s rank coefficient between FA and the concentration of trace metals in needles did not show any significant correlation in both groups. However, in group S, there was a very strong negative correlation between FA and Cu (*r* = −0.91) and a medium negative correlation between FA and Fe (*r* = −0.44). There also was a medium positive correlation between FA and Mg (*r* = 0.46). The remaining correlations, between FA and Zn, Pb, and Cd, are negative and very weak. In both groups S and T, there are very strong negative correlations between FA and Mg (*r* = −0.90) and FA and Fe (*r* = −0.89) and strong positive correlations between FA and Pb (*r* = 0.77), FA and Cd (*r* = 0.72), and FA and Zn (*r* = 0.62). Between FA and Cu, there is a medium positive correlation (*r* = 0.40).

**Table 3:** The comparison of FA index within and between S and T group

Year	Mean of FA index ± SE		Mann-Whitney *U* test
	S	T	
2006	0.0306 ± 0.004	0.0043 ± 0.001	*U* = 46.5***
2010	0.0429 ± 0.005	0.0041 ± 0.001	*U* = 9.5***
2014	0.0426 ± 0.005	0.0039 ± 0.002	*U* = 2.0***
*Grand mean*	0.0387 ± 0.003	0.0041 ± 0.002	*U* = 199***
Friedman test (*χ* ^2^)	2.46 ns	2.82 ns	

*ns* not significant value, *SE* standard error

*** Value significant on 0.001

**Fig. 4:**
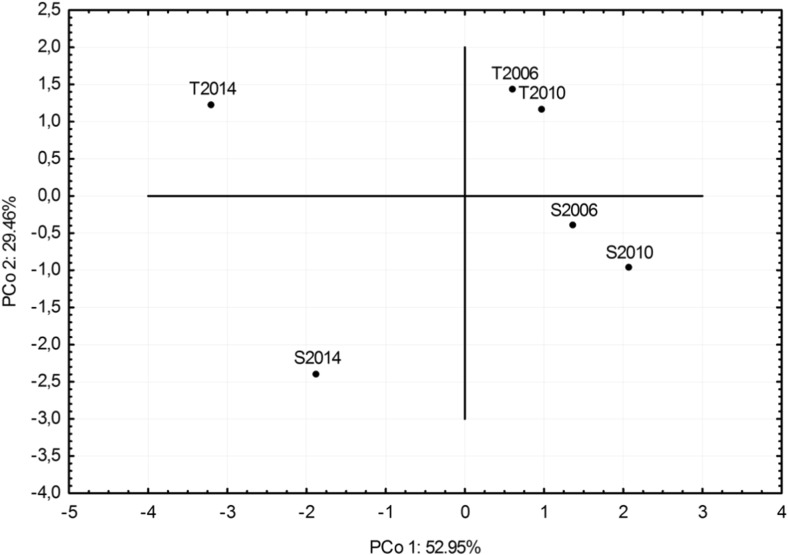
Principal coordinates analysis (*PCA*) made on the basis of the FA index, concentration of trace metals, and observed heterozygosity

The principal coordinates analysis (PCoA) of the FA index, concentration of trace metals, and observed heterozygosity (Fig. 4) showed that for the years 2006 and 2010, we may distinguish two groups individually from groups S and T. Data from the year 2014 differs significantly from the remaining years and, having shown the clearest division into the S and T groups of trees. The correlation coefficient between individual heterozygosity (*H*_ind_), FA, and concentration of trace metals in needles did not show any significant correlation in group S. In group T, there was a significant, negative, and medium correlation between *H*_ind_ and the concentration of zinc (*r* = −0.39, *p* = 0.049).

### Genetic diversity parameters

**Fig. 5:**
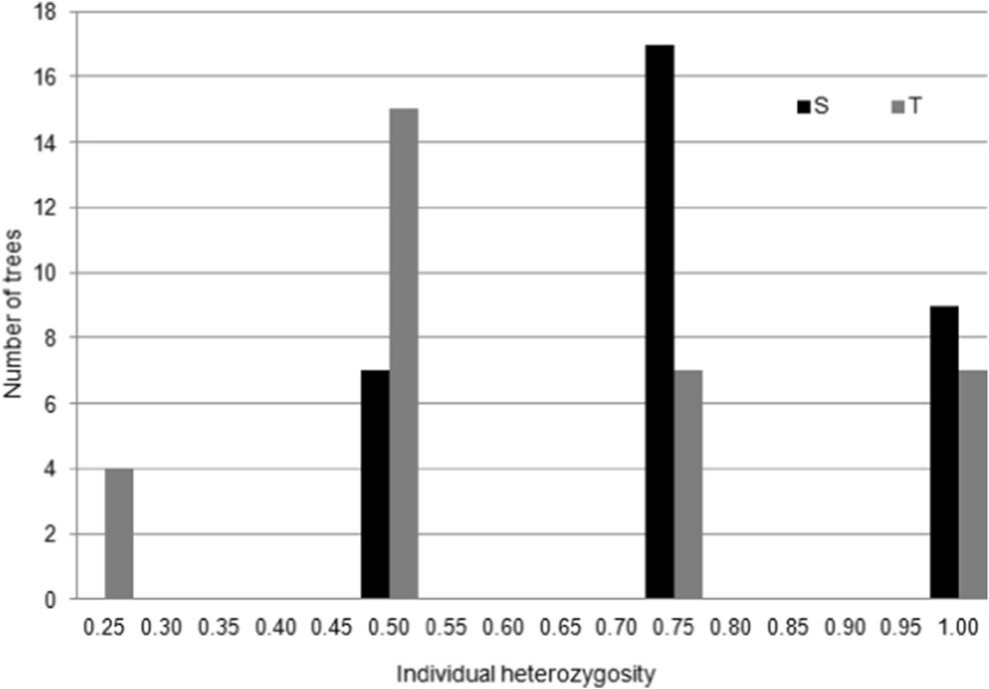
Frequency distribution of individual heterozygosity (*H*_ind_) for the S and T group of trees

Four analyzed nuclear microsatellites were polymorphic. A total of 51 alleles were found in the group of sensitive trees (S) and 52 alleles in the group of tolerant trees (T). There were no significant differences among the sensitive (S) and tolerant (T) groups of trees in the total number of alleles, the average number of alleles per locus, or in the average number of effective alleles (Table 4). Significant differences between the S and T groups were noticed in particular SSR loci with regard to the number of alleles (*N*_A_), i.e., 25 alleles were found in locus SPAG 7.14 in group S while only 18 alleles were noticed in group T. In the three other microsatellite loci, a higher number of alleles were detected in the T group than in the S group (Table 4). The number of private alleles (*N*_AP_) was highly variable among particular SSR loci and the groups (Table 4). The highest number of private alleles was noticed in the S group for SPAG 7.14 (9) and the lowest (1) for PtTX 4001 and PtTX 4011 in the same group of trees. In the T group, there were six private alleles in PtTX 4011, five alleles in PtTX 4001, and two alleles in SPAG 7.14 and SPAC 11.4. The observed heterozygosity (*H*_O_) varied between loci and the two analyzed groups of trees (Table 4). There were no significant differences in the average value of the observed heterozygosity among the S and T groups. High differences were observed in the average fixation index value (*F*), which amounted to 0.057 for the S group and 0.179 for the T group. The Hardy-Weinberg test (HWE) indicated that in the S group, all SSR loci were in equilibrium, while in the T group three of the four SSR loci were not. The Mann-Whitney *U* test revealed significant differences in the individual heterozygosity (*H*_ind_) values (Fig. 5) of particular trees from the S and T tree groups (*p* = 0.0136). An analysis of molecular variance (AMOVA) revealed that 2 % of total genetic variance was present among the sensitive and tolerant tree groups, while the remaining 98 % was observed within the Scots pine groups (Table 5).

**Table 4:** Genetic parameters and differentiation of the sensitive (S) and tolerant (T) groups of Scots pine trees growing in trace metals polluted soil

Group of trees^a^	SSR marker	*N*_A_	*N*_E_	*N*_AP_	*H*_O_	*H*_E_	*F*	HWE
Sensitive (S)	SPAG 7.14	25	15.9	9	0.788	0.937	0.159	ns
	PtTX 4011	6	3.6	1	0.576	0.719	0.199	ns
	PtTX 4001	7	3.9	1	0.818	0.741	−0.105	ns
	SPAC 11.4	13	7.0	3	0.879	0.858	−0.024	ns
	Mean	12.75	7.6	3.5	0.765	0.814	0.057	
	SD	4.37	2.9	1.9	0.066	0.051	0.073	
Tolerant (T)	SPAG 7.14	18	11.5	2	0.606	0.913	0.336	**
	PtTX 4011	11	2.6	6	0.455	0.611	0.256	***
	PtTX 4001	11	3.9	5	0.606	0.745	0.187	**
	SPAC 11.4	12	5.0	2	0.848	0.799	−0.062	ns
	Mean	13.0	5.8	3.8	0.629	0.767	0.179	
	SD	1.68	2.0	1.0	0.081	0.063	0.086	
Genetic differentiation^b^	SPAG 7.14	PtTX 4011		PtTX 4001		SPAC 11.4		Total
Ф_PT_	−0.005	0.067*		0.000		0.011		0.017*

^a^Genetic parameters (genetic diversity indices) of sensitive (S) and tolerant (T) group of Scots pine trees growing in heavy metal polluted soil. *N*_*A*_ number of alleles, *N*_*E*_ number of effective alleles, *N*_*AP*_ number of private alleles, *H*_*O*_ heterozygosity observed, *H*_*E*_ heterozygosity expected, *F* fixation index, *HWE* Hardy-Weinberg equilibrium test (*ns* not significant. *p < 0.05; **p < 0.01; ***p < 0.001)

^b^The genetic differentiation (Ф_PT_) of sensitive (S) and tolerant (T) group of Scots pine trees growing in heavy metal polluted soil in particular SSR loci. *p < 0.05; **p < 0.01; ***p < 0.001

**Table 5:** Analysis of molecular variance (AMOVA) within/among the sensitive (S) and tolerant (T) groups of Scots pine trees growing in trace metals polluted soil

Source	df	SSD	MSD	Variance components	Total variance (%)	*P* ^a^
Among S and T groups	1	5.742	5.742	0.064	2	<0.001
Within groups	64	233.333	3.646	3.646	98	

^a^ *P* values are the probabilities of having a more extreme variance component than observed values by chance alone. Probabilities calculated by 999 random permutations of individuals across populations

## Discussion

### Trace metals phytoaccumulation—dynamics and species-based trends

The analyses performed at regular intervals of time should reflect the relationships between changes in the trees’ condition and changes to their nutritional status. The process of trace elements accumulation in plants and the resulting impacts on mineral nutrient concentrations depend on two basic factors: (i) genetic and biological characteristics, and (ii) mineral levels of the growth medium and abiotic pressure. Both factors interact strongly and shape what we express as the environmental bio-mineral adaptability. The case of the Scots pine, as reported in Table 2, deserves particular attention. The investigated trees are approximately 40 years old and have been exposed since germination to an extremely strong environmental contamination pressure. Lead, zinc, and cadmium contents were mostly of great concern caused by high soil concentrations. Data revealed that Cu, Zn, Pb, and Cd concentrations in the C + 1 old needles were significantly higher in the case of sensitive as compared to tolerant groups. The accumulation pattern follows consequently the sequence, i.e., Pb > Cd > Cu > Zn representing in relative values 117.4 > 67.3 > 62.9 > 27.8 %.

The first two metals are chemically toxic as compared to Cu and Zn, which exhibit phytotoxicity when their concentrations exceed a given threshold. The reference values, as suggested by Markert (1994) and Pais and Benton Jones (1997), reveal that both investigated pine types accumulated high to extremely high concentrations of all trace metals. The assessment of the potential accumulation intensity was undertaken by establishing the sequences of the quotients of mean Cu, Zn, Pb, and Cd concentrations to the reference values (Table 2). This approach resulted in the following arrangements:

Tolerant pines: Pb (258) > Cd (196) > Zn (7) > Cu (1)

Sensitive pines: Pb (560) > Cd (328) > Zn (9) > Cu (1.5)

Lead and cadmium are the metals that were much more preferably accumulated by the C + 1 old pine needles. Next, their mobility in plants is strongly contrasting with Pb being less mobile (slow translocation), whereas Cd (similarly to Zn) experiences the opposite. The oldest needles may have developed a physiological translocation barriers of assimilates, making the phytotoxicity of the reported metals seem unavoidable. This process shapes and regulates survival reactions, which in turn strengthen the accumulation of vital elements—particularly Mg and Fe within the Scots pine.

### Mineral-survival strategies—Mg and Fe rescue

It has been evident for a long time that magnesium, as well as iron, plays a key role in the processes of plant photosynthesis; they serve a vital plant mechanism, irrespective of growth conditions (Helmisaari 1992; Huettl,^1993^; Rautio 2000). Next, several factors—some biotic but mostly abiotic (light intensity, mineral status in the growth medium, stressors, among others)—were identified as crucially responsible for chlorophyll production and maintenance. Needles, as in the case of *P. sylvestris*, exhibit a specific morphology; for example, reduced leaf area, which in turn is highly efficient in light interception for active photosynthesis. Therefore, we have formulated the hypothesis that the concentrations of both Mg and Fe in Scots pine needles should serve as a physiological-toxicological index for evaluating trace metals “lethality.”

This concept was verified on the basis of the relationships reported in Fig. 1 (for tolerant Scots pine trees) and Fig. 2 (for sensitive Scots pine trees), where Pb, Cd, and Zn concentrations in needles were regressed against Mg and Fe concentrations. The choice of only three trace metals was dictated by the values of the potential accumulation intensity (PAI). The basic findings that should result from Figs. 1 and 2 are related to the effects (impacts) of these trace metals on Mg and Fe accumulation in the C + 1 old needles, irrespective of their health status. The course of these effects should be stressed, as they are manifested linearly. This implies that Mg and Fe are elements controlling the survival of Scots pines, particularly under trace metals pollution. Briefly, the higher Zn, Pb, and Cd concentrations in the needles results in the highest levels of Mg and Fe accumulation. A rough assessment of these reactions–responses (R-R) is undertaken via regression analysis (Figs. 1 and 2).

Two R-R patterns are evident: (i) Mg with the highest *R* ^2^ values particularly for Cd and Zn, (ii) Fe with the highest *R* ^2^ values particularly for Pb and Cd. The key findings of these patterns confirm the process of mineral mobility with plant assimilates. Magnesium is very mobile, as are Cd and Zn, in contrast to Fe and Pb (Pais and Benton Jones 1997), but both, i.e., tolerant as well as sensitive trees, have exhibited responses to Cd. This is indicative of the acute toxicity of this metal, irrespective of the impacted individual. These data correspond to the results of Pietrzykowski et al. (2014), which have demonstrated that in soils located in the Upper Silesia region, the concentration of Cd in pine needles is higher in post-mine ecosystems due to pollutant deposition.

### Relationship between metal concentration and FA

A number of studies have confirmed that FA tends to increase in response to adverse conditions, so this parameter is frequently used in ecological studies as an indicator of the stress experienced by an organism due to environmental pollution (Graham et al. 2010). We have to remember that FA is characterized by the same unspecific symptoms such as discoloration, height, or increased crown defoliation, and only manifests the general health status of trees, without an indication of the nature of the stress agents. For the C + 1 old needle Scots pine growing near the zinc smelter, the FA measurement has enabled an evaluation of tree health conditions. During 8 years of observation, we noted the same tendencies in FA indices, dividing trees into two groups: sensitive and tolerant to pollution. This was evident even when the contamination level expressed in the trace metals content was significantly reduced (for the year 2014). It is probable that in sensitive trees, adaptive modifications must buffer the stress to such an extent that the FA is a reflection of the fact that the stress exceeded an organism’s homeostatic abilities and affected the stability of its development. A similar reaction was demonstrated in several plant species. Mal et al. (2002) found that the FA of the wetland species *Lythrum salicaria* increased in a lead-polluted environment versus a non-polluted one. Kozlov et al. (2009) found that the FA of two birch species, *Betula pendula* and *Betula pubescens*, decreased with increasing distance from copper-nickel smelters and were positively correlated to foliar nickel concentrations. Relationships between individual fluctuating asymmetry and fitness with other morphological and anatomical measures were correlated to a lesser extent (Chudzińska et al. 2014b). We note that FA is a more sensitive indicator of stress than particular morphological changes. These changes evolve in the adaptation process to reduce the effects of stress, while FA is not an adaptation, but rather a “symptom” of stress. Interestingly, according to the results of studies on different organisms, the loss of genetic variation may cause increased fluctuating asymmetry, especially in populations subjected to bottlenecks, or rapid environmental changes caused by various stresses (Leamy and Klingenberg 2005). A deficiency in genetic variance leads to an increase in the homozygosity of individuals. Because different alleles may have different optima for producing their enzymatic products, heterozygotes are considered to be better adapted because they maintain adequate levels of enzymatic activity over a wider range of environmental variation than do homozygous individuals. According to this assumption, heterozygous individuals are less sensitive to environmental variables than homozygous ones, and, supposing that the enzyme activity is related to the measured characters, homozygous individuals may show increased levels of fluctuating asymmetry when compared to heterozygous ones. In our data, we observed an opposite reaction: the mean value of observed heterozygosity (*H*_O_) was higher in group S (0.765), and the test for Hardy-Weinberg proportions showed highly significant (p < 0.001) heterozygote deficiency in tolerant trees (T) in respect to three loci. This can be interpreted as neutrality of SSR markers, but similar results were obtained in studying enzyme variation. A higher *H*_O_ value in roughly half of the analyzed loci in *P. sylvestris* was higher in the sensitive tree group (Chudzińska et al. 2014a). These data suggest that better fitness (i.e., lower FA) is likely a sign of adaptation to a severe level of pollution for only a narrow group of specialized individuals.

### Relationship between metal concentration and genetic diversity

According to Dickinson et al. (1991), the longevity of trees prevents the fast selection of trace metals tolerant genotypes stimulated by contamination. Consequently, trees are considered to be unable to adapt to high concentrations of trace elements in the soil. However, microsatellite DNA results showed that pollution-sensitive and pollution-tolerant Scots pine subpopulations differ genetically from each other. As Ernst (2006) underlined, it was proved that natural selection induced by adverse environmental conditions may cause increased differentiation between tolerant and non-tolerant populations. The description of these differences is made on the basis of the parameters used in population genetics, such as the number of alleles and the effective number of alleles in particular loci, the number of private alleles, observed heterozygosity, individual heterozygosity, the value of the fixation index and Hardy-Weinberg deviations, etc. The mean effective number of alleles and mean observed heterozygosity were respectively 24 and 18 % lower in the group of pollution-tolerant trees. These differences are even more pronounced in the particular loci, especially in locus PtTX4001. The distribution of the individual heterozygosity of particular trees in both S and T groups also confirmed their genetic differentiation. AMOVA indicated that most of the genetic variation was due to differences among individuals within Scots pine subpopulations, although 2 % of variance was found between S and T groups. This value is generally comparable with the data published previously by Nowakowska (2007) for 42 Scots pine populations located in 6 Natural-Forest Regions in Poland using microsatellite DNA markers (*F*_ST_ = 0.03). Therefore, the 2 % genetic differentiation among the two groups of trees (from the same localization) found in our study may indicate the strong selective pressure that leads to the differentiation of the local population. Pollution-sensitive tress appeared to be in Hardy-Weinberg equilibrium, while the pollution-tolerant subpopulation, exhibiting a highly adapted genotype to this specific polluted habitat, had a significant excess of homozygotes in three out of four analyzed loci.

Since SSRs have been widely applied to the process of elucidating genetic diversity and population structure in plants, there is a discussion over their suitability for this type of research (Li et al. 2002). These marker systems are assumed to be selectively neutral. However, increasingly more studies show that there is a link between SSRs and adaptation to different environmental conditions (Rocha et al. 2002). The most likely explanation for this relationship is a hypothesis that we are dealing with here: the phenomenon of genetic “hitchhiking” (Ribeiro and Lopes 2013). The SSR loci closely related to advantageous alleles might explain any shifts from neutral expectations. According to Allendorf et al. (2010), even a small proportion (1–5 %) of non-neutral loci can change the estimates of the mean *F*_ST_ values by 30–50 %. In our studies, the excess homozygosity at these loci looks to be fitness related (either directly, which is unlikely for microsatellite loci, or due to the physical linkage of the marker with a trait locus). Tolerant trees demonstrated a lower degree of genetic variation than pollution-sensitive trees with respect to some nuclear microsatellite loci. This is in agreement with previous studies conducted on the same polluted site using isoenzyme markers (Prus-Głowacki and Nowak-Bzowy 1992; Chudzińska et al. 2014a). A similar loss of genetic variability was observed in metallicolous populations of *Sedum alfredii* Hance, *Armeria maritima*, and *Silene paradoxa* (Deng et al. 2007). Similar results were obtained by Mussali-Galante et al. (2013) for *Peromyscus melanophrys*, showing a significant negative relationship between genetic diversity and the metals Al, Pb, Cu, As, and Cd concentrations. Such an adaptation strategy in a heavily contaminated environment leads to a decrease in the level of genetic variation, and consequently to the narrowing of the gene pool. In conclusion, these results suggest that genetic diversity measured by means of the nuclear microsatellite markers of the Scots pine population living near the trace metals smelter is affected by trace metals exposure. Despite the fact that in different years, the high variability of the tested parameters has been observed, the division of trees into S and T groups on the basis of individual heterozygosity (*H*_ind_), FA, and concentration of trace metals remained unchanged.

## Conclusions

Strong trace metals pollution induced significant changes both on the phenotype of the individual tree as well as on the genetic structure of the *P. sylvestris* population. The trees that are growing near the zinc smelter responded in a different way to their exposure to trace elements, which is probably a result of their varied genetic constitution. The observed decrease in Cu, Zn, Pb, and Cd concentrations in C + 1 needles over the years of investigations is associated with an increase in Mg and Fe levels. These two groups of mineral elements seem to be the key factors affecting the growth of the pine needles under the conditions of metallurgical pollution. We have formulated a hypothesis that the concentrations of both Mg and Fe in the Scots pine needles should be considered a physiological-toxicological index for evaluating trace metals “lethality” expressed as Mg and Fe mineral-survival strategies.

In the case of *P. sylvestris*, fluctuating asymmetry (FA) is a good indicator of environmental stress, and needles of pollution-tolerant (T) and pollution-sensitive (S) individuals exhibited different levels of metal accumulation. The genetic structure of the observed populations exposed to trace elements may be altered by a reduction of their genetic variation, e.g., through selective sweeps which affect the frequency of alleles. If so, changes in allele frequencies may ultimately lead to an adaptation of the populations to the environmental pollution. In the case of the Scotch pine, a high level of pollen migration from the neighboring population, normal in the case of wind-pollinated species, can restore the initial level of variation in the populations exposed to pollution.

In biomonitoring, the selection of examined individuals must be based on the rigid criteria of spatial representativeness. During specimen observation, correct data have to take into account not only the underlying measuring or sampling methodology but also an individual response reaction to stress conditions. Currently, incorporating measurements of genetic diversity for diagnosing the state of environmental pollution is not frequent, but this could change, especially when new data confirm with greater precision the observed interdependencies. These studies show one of the aspects of the correlations between high trace metals contamination and the genetic differentiation of Scots pine populations growing in the polluted environment.
